# A Case of Pedunculated Adenoma of the Bile Duct Proving Adenoma to Cancer Sequence

**DOI:** 10.7759/cureus.58304

**Published:** 2024-04-15

**Authors:** Mohammed Ahsan, Medjine Jarbath, Brittany Davis, William J Provance

**Affiliations:** 1 Graduate Medical Education, Naples Community Hospital Healthcare System, Naples, USA; 2 Gastroenterology and Hepatology, Naples Community Hospital Healthcare System, Naples, USA

**Keywords:** ampulla of the vater, tumors of the ampulla, endoscopic ultrasound (eus), sepsis, cholangitis, adenocarcinoma, adenoma to carcinoma sequence

## Abstract

Ampullary adenocarcinoma is a rare malignancy that originates in the ampulla of Vater. It typically presents in the seventh decade of life. This condition shares overlapping features with periampullary tumors such as pancreatic cancer, but treatment modalities and prognosis vary. Histology will demonstrate either intestinal or pancreato-biliary epithelial subtype in ampullary adenocarcinoma. Despite its rare occurrence, ampullary adenocarcinoma should be included as a differential in elderly patients presenting with biliary obstruction. This case presentation is unique as it highlights the importance of histopathological findings and their progression. In this case, initial histology results revealed tubulovillous adenomatous polyps, but later biopsies revealed adenocarcinoma cells. These findings suggest that ampullary adenocarcinoma and several gastrointestinal cancers share a similar mechanism of action as it is related to the adenoma-to-carcinoma sequence. This case presentation aims to highlight the rare occurrence of this phenomenon at the ampulla of Vater.

## Introduction

Ampullary adenomas are precancerous lesions that result from mucosal overgrowth of the ampulla [1]. They may sporadically form or be associated with hereditary polyposis syndromes such as familial adenomatous polyposis [2]. The risk of developing ampullary malignancies in patients with polyposis syndromes is as high as a 300-fold increase compared to the general population [3]. Thus, patients who have hereditary syndromes present much earlier with ampullary tumors compared to patients with sporadic form, which is typically diagnosed among patients in their sixth-seventh decade of life [4]. Patients may be asymptomatic or present with mass effects. Symptoms may progress and be consistent with common bile duct obstruction and cholangitis. Diagnostic modalities include endoscopic retrograde cholangiopancreatography (ERCP), abdominal computed tomography (CT), magnetic resonance cholangiopancreatography (MRCP), and endoscopic ultrasound (EUS). Imaging modalities play an integral role in diagnosis. Confirmation of the diagnosis requires biopsy and histopathology [5]. Histopathology will further demonstrate a biliopancreatic or intestinal tumor type stemming from the papillary mucosa [6]. We present a case of concurrent adenocarcinoma with tubulovillous adenoma with high-grade dysplasia highly suggesting the progression of ampullary adenoma to ampullary carcinoma.

## Case presentation

**Figure 1:**
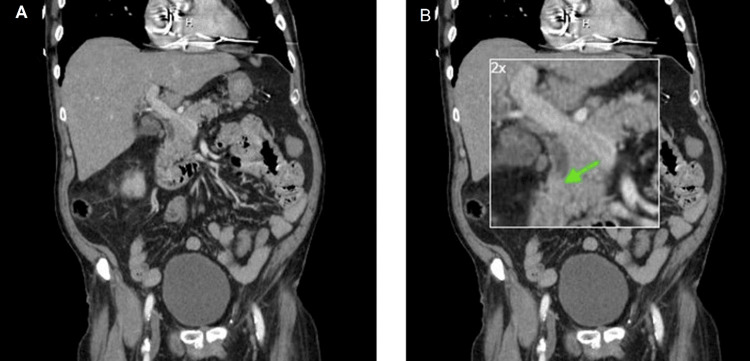
CT abdomen and pelvis (coronal) demonstrating (A) distal bile duct filling defect (green arrow) and (B) with 2x magnification

An 83-year-old male with a past medical history of coronary artery disease status post coronary artery bypass graft, chronic renal disease stage 3b, non-insulin-dependent type 2 diabetes mellitus, pacemaker, and atrial fibrillation anticoagulated with apixaban, presented with weakness, emesis, and right upper quadrant/epigastric pain. A review of the systems was positive for fevers and chills. His symptom onset was three days before admission. Vital signs were only remarkable for hypertension with a blood pressure of 166/64 mmHg (normal range is under 130/90). Laboratory results were remarkable for blood urea nitrogen (BUN) 34 mg/dl (normal range is 6-21 mg/dl), creatinine 1.6 mg/dl (patient's baseline creatinine around 1.1 mg/dl), with no leukocytosis on admission 8.2 10^3^/uL (4.2-10.8 10^3^/uL) but increased to 13 10^3^/uL (4.2-10.8 10^3^/uL) the following day. The hepatic function panel was remarkable for elevated alkaline phosphatase 372 U/L (normal range is 50-136 U/L), low albumin 2.9 g/dl (3.4-5 g/dl), elevated total bilirubin 1.6 mg/dl (0.2-1.0 mg/dl), elevated direct bilirubin 0.44 mg/dl (0-0.2 mg/dl), aspartate aminotransferase (AST) 273 U/L (normal range is 15-37 U/L), and alanine transaminase (ALT) 146 U/L (normal range is 13-61 U/L). CT abdomen with intravenous contrast was notable for a dilated common hepatic duct at 9.5 mm and distal bile duct at 10 mm. A filling defect was seen in the distal bile duct (Figure 1). The gallbladder ultrasound showed intrahepatic and extrahepatic ductal prominence secondary to a 2 cm echogenic focus within the lumen of the distal common bile duct at the level of the pancreatic head. Blood cultures were positive for *Klebsiella pneumoniae*. Despite normal vital signs, there was a concern for low-grade cholangitis. The patient was started on empiric antibiotics (cefepime and metronidazole) and underwent an ERCP.

**Figure 2:**
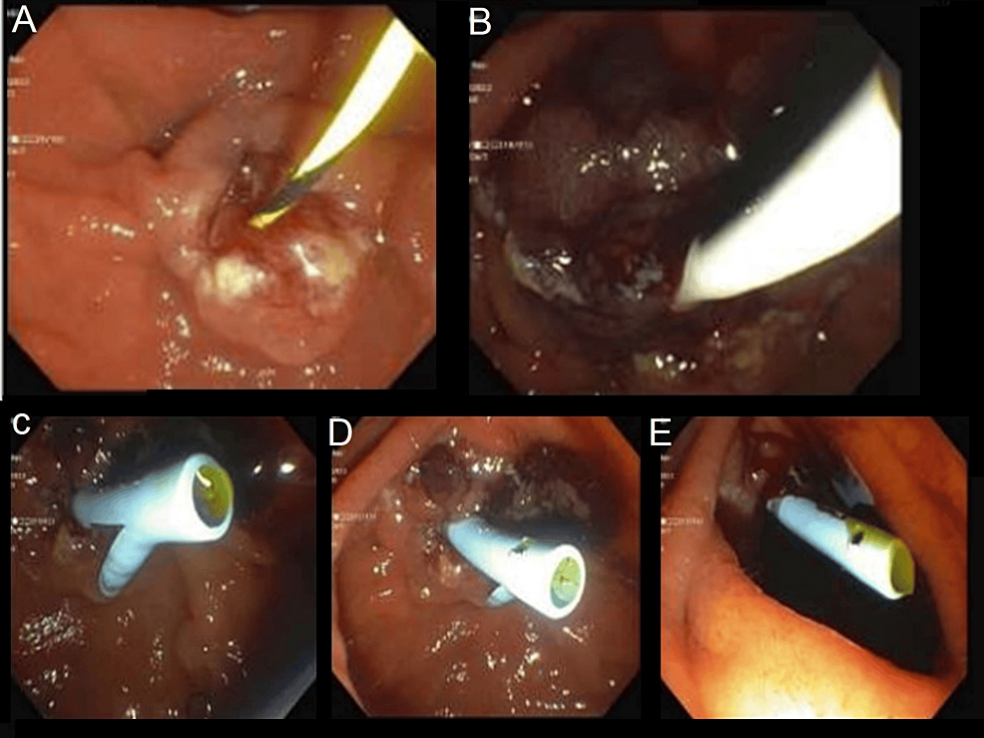
A polyp (abnormal growth) can be seen in the ampulla, which prolapses out into the duodenum with the balloon sweep. Grossly, it appears as grapelike clusters on endoscopy. Surrounding papilla appears normal (A) Area of papilla in the duodenum; (B) Area of papilla in the duodenum, probable adenoma; (C) Area of papilla in the duodenum; (D) Prolapsed adenomatous tissue; (E) Area of papilla in the duodenum

The ERCP revealed the major papilla was located entirely within a diverticulum in a pantaloon configuration. The papilla itself appeared normal. The bile duct was deeply cannulated. A filling defect was seen on the cholangiogram at the ampulla extending into the lower bile duct. The middle third of the common bile duct was moderately dilated. A sphincterotomy was performed and a grape-like polyp prolapsed into the duodenum with a balloon sweep (Figure 2). Biopsies revealed tubulovillous adenoma with high-grade dysplasia. A temporary plastic stent was then placed into the common bile duct for appropriate drainage.

**Figure 3:**
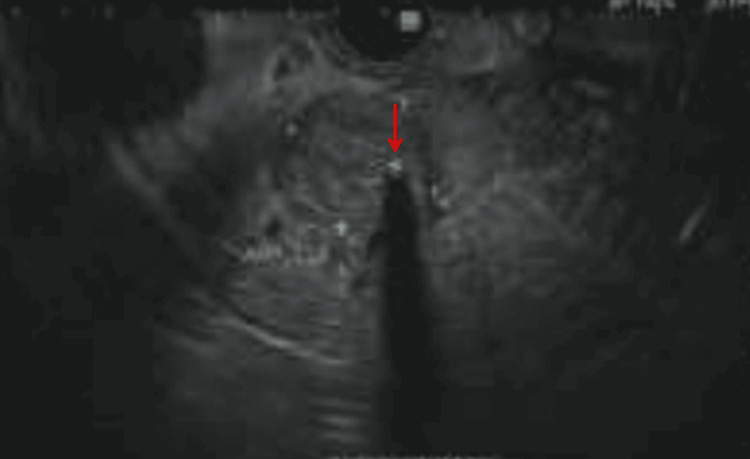
On endoscopic ultrasound, a hypoechoic oval mass (red arrow) with post-acoustic shadowing was identified endosonographically in the ampulla, measuring 16 mm by 15 mm in cross-sectional diameter.

Due to concerns for invasive ampullary adenocarcinoma, an EUS was performed. This revealed a hypoechoic mass in the ampulla measuring 16 mm by 15 mm in cross-sectional diameter (Figure 3). Fine needle biopsy showed atypical glandular cells with increased nuclear to cytoplasmic ratio and prominent nucleoli consistent with adenocarcinoma. Given that the patient’s cultures were found to be sensitive to amoxicillin/clavulanate, the patient was transitioned to augmentin for two weeks on discharge.

After discharge, the patient was established with a medical oncologist, with plans to start chemotherapy with capecitabine along with radiation therapy for six weeks. Following radiation therapy, the patient was going to establish with a surgical oncologist to discuss options for Whipple surgery.

## Discussion

Ampullary carcinoma is a rare malignancy with an incidence rate of only 0.2% [7]. Unlike other gastrointestinal tumors such as colon, gallbladder, and pancreatic cancer, ampullary carcinomas are associated with a better prognosis, emphasizing the significance of early diagnosis [8]. The conversion from adenoma to carcinoma, however, is rare [9]. In this case, a patient with no prior personal or family history of hereditary polyposis syndromes or hereditary nonpolyposis colorectal cancer presented with low-grade cholangitis and gram-negative bacteremia secondary to a localized polypoid ampullary adenocarcinoma.

EUS subsequently revealed a hypoechoic mass in the ampulla measuring 16 mm by 15 mm. Biopsy results revealed atypical glandular cells with increased nuclear to cytoplasmic ratio and prominent nucleoli consistent with adenocarcinoma. Although rare, these findings are highly suggestive of a stepwise progression from normal cellular histology to dysplastic epithelium with further progression to carcinoma. An additional caveat to this case is that most facilities do not have EUS capabilities. Having the capability to perform EUS facilitated the relief of the obstruction and identified the mass obstructed by the polyp in the ampulla. This finding was pivotal and led to a referral to the surgical oncology team for Whipple’s procedure. This case also highlights the need for routine visualization of the ampulla of Vater during esophagogastroduodenoscopy, especially in individuals presenting with biliary obstruction. As this topic continues to be studied it should be noted that certain types of scopes are ideal for the visualization of the ampulla of Vater. Some studies suggest that using the standard forward-viewing cameras on endoscopes allows visualization of the ampulla of Vater approximately 70.9% of the time, but how often this is reliably seen is unclear [10].

Given that ampullary carcinomas are rare and associated with a high five-year mortality rate, it is important to consider this condition in elderly patients presenting with an obstructed common bile duct. Furthermore, differentiating ampullary adenocarcinoma from other malignancies is paramount. If discovered early enough, surgery can be curative in 50% of cases of ampullary adenocarcinoma compared to 10% of cases of pancreatic adenocarcinoma [11]. Unfortunately, the rate of recurrence in surgically treated patients with ampullary carcinoma remains at 45% [12]. Therefore, the prognosis remains guarded, rendering management for this condition a challenge. Further research is thus required to increase survival in this subgroup of patients.

## Conclusions

This case demonstrates a unique presentation of an ampullary polyp that subsequently led to obstructive cholangitis while masking an underlying malignant mass. This case highlights several important principles, including how the adenoma-carcinoma sequence applies to lesions in the ampulla, and therefore special attention should be given to this region during screening endoscopies and colonoscopies. In our case, EUS also allowed visualization of a malignant mass that was obstructed by a tubulovillous adenomatous polyp on the ampulla, significantly affecting management in our patient. Thus, the utility of EUS cannot be understated, as it allows for better visualization of the underlying anatomy and the ability to obtain fine needle biopsies for diagnostic and staging purposes. 
